# Access Site Selection and Outcomes for Chronic Total Occlusion Percutaneous Coronary Interventions: Insights from the VA CART Program

**DOI:** 10.1016/j.jscai.2022.100440

**Published:** 2022-08-24

**Authors:** Jaidip Chakravartti, William J. Feser, Mary E. Plomondon, Javier A. Valle, Sunil V. Rao, J. Antonio Gutierrez, Gary K. Grunwald, Elise Gunzburger, Rajesh V. Swaminathan

**Affiliations:** a Department of Cardiology, Maine Medical Center, Portland, Maine; b Rocky Mountain Regional VA Medical Center, Aurora, Colorado; c Department of Biostatistics and Informatics, Colorado School of Public Health, University of Colorado, Aurora, Colorado; d CART Program, Office of Quality and Patient Safety, Veterans Health Administration, Washington, DC; e Section of Cardiology, University of Colorado School of Medicine, Aurora, Colorado; f Department of Medicine, Division of Cardiology, Duke University School of Medicine, Durham, North Carolina; g Duke Clinical Research Institute, Durham, North Carolina; h Section of Cardiology, Department of Medicine, Durham VA Healthcare System, Durham, North Carolina

**Keywords:** chronic total occlusion, percutaneous coronary intervention, transfemoral, transradial

## Abstract

**Background:**

There has been increasing use of transradial access (TRA) for non–chronic total occlusion (CTO) percutaneous coronary intervention (PCI). There are limited data on TRA for CTO PCI. The objectives of this study were to assess the temporal trends in the use of TRA versus transfemoral access (TFA), identify procedural and lesion characteristics associated with the use of TRA and TFA, and evaluate the association of access site with procedural complications and technical success among veterans undergoing attempted CTO PCI.

**Methods:**

We performed a retrospective analysis of veteran patients who underwent attempted CTO PCI to compare outcomes between TRA and TFA. Patients who had undergone attempted PCI of at least 1 CTO were included. Propensity score matching was used to evaluate the composite primary outcome of major procedural complications, in-hospital bleeding, or 30-day mortality and the secondary outcome of procedural success.

**Results:**

In total, 4609 patients underwent attempted CTO PCI during 2010-2017. Rates of TRA for CTO PCI increased significantly, from 7% in 2010 to 38% in 2017 (*P*_trend_ < .01). A greater percentage of CTO lesions in the TFA group was calcified and >20.0 mm in length. TRA was not associated with a reduction in the composite primary outcome (TRA 3.3% vs TFA 4.0%, *P* = .47) or procedural success (TRA 66.6% vs TFA 65.7%, *P* = .74) compared with TFA.

**Conclusions:**

In this retrospective analysis of patients who underwent attempted CTO PCI, the proportion of TRA for CTO PCI has increased over time but was not associated with a greater safety or procedural success than TFA.

## Highlights

• Retrospective study of veterans who had intervention for chronic total occlusion.
• Increased use of transradial access over the study period.
• Similar safety and technical success rates for transradial and transfemoral access.

## Central Illustration

**Figure:**
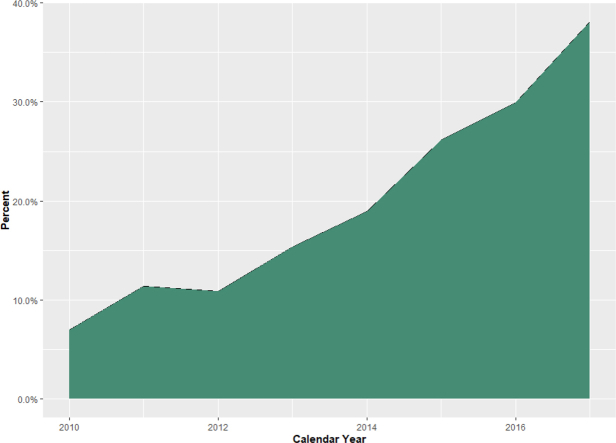

## Introduction

Over the past decade, transradial access (TRA) has become increasingly used for percutaneous coronary intervention (PCI).^1^, ^2^, ^3^ This trend has been driven primarily by lower rates of access site complications, major bleeding, transfusion, and mortality with TRA compared with those of transfemoral access (TFA).^4^, ^5^, ^6^, ^7^, ^8^, ^9^, ^10^, ^11^ However, TFA has historically been the preferred access route for chronic total occlusion (CTO) PCI because of frequent use of large caliber guide catheters that can accommodate devices used to facilitate CTO lesion crossing and to provide additional support.^12^, ^13^, ^14^

Advances in the design of CTO-crossing devices, coronary guide wires, larger bore sheathless transradial guide catheters, and guide catheter extensions provide opportunities for increased use of TRA for CTO PCI.^15^, ^16^, ^17^ In addition, because dual arterial access is commonly necessary for collateral visualization, TRA can be used for this purpose rather than a second TFA.

Although a growing body of evidence highlights the potential role of TRA for CTO PCI, these data are limited to single-center experiences or registry-based studies from extremely high volume CTO centers.^18^, ^19^, ^20^, ^21^, ^22^, ^23^, ^24^, ^25^, ^26^, ^27^, ^28^ This study used contemporary multicenter Veterans Health Administration data to: (1) assess trends in access site selection for CTO PCI, (2) identify procedural and lesion characteristics associated with the use of TRA versus TFA, and (3) evaluate the effect of access site selection on procedural complications and technical success. We hypothesized that rates of TRA for CTO PCI would increase over the study period while maintaining technical procedural success and that TRA would be associated with fewer procedural complications.

## Methods

### Data sources

For this study, we used data from the Veteran Affair (VA) Clinical Assessment, Reporting, and Tracking (VA CART) program. The CART program uses standardized definitions for procedural variables derived from the National Cardiovascular Data Registry.^29^^,^^30^ For every invasive cardiac procedure performed in the VA system, the CART application is used to collect associated clinical and procedural data. CART data are combined with other VA data sources to obtain baseline patient characteristics, such as demographic information, comorbidities, medications, echocardiographic data, laboratory values, and longitudinal outcomes.^31^ The Colorado Multiple Institutional Review Board approved a waiver of informed consent for this study.

### Study sample and design

Patients were included in the analysis if they had undergone attempted CTO PCI between January 1, 2010, and December 31, 2017. Only index attempts at revascularization of a specific CTO lesion were included. In the CART database, CTO was identified by meeting 1 of the following criteria: operator-identified CTO or having 100% prestenosis with the primary indication for PCI being stable angina. Patients were excluded for any of the following reasons: if the primary PCI indication was acute coronary syndrome, ST elevation myocardial infarction (MI), non-ST elevation MI or unstable angina, concomitant cardiogenic shock, missing access site information, single access site without femoral or radial access, or dual access sites without any femoral or radial access. The index CTO PCIs that met the overall inclusion and exclusion criteria were included in the temporal trend cohort. A subset of the temporal trend cohort, the analytic cohort, was used to analyze the outcomes. The analytic cohort excluded CTO PCIs that were missing the primary indication or creatinine. CTO PCIs were also excluded from the analytic cohort if they were performed at VA sites that had >90% radial access, >90% femoral access, or <10 attempted CTO PCIs in the study period.

For the purpose of analysis, if at least 1 radial access site was used, the patient was categorized as the TRA group. If 1 radial and 1 femoral access points were used, the patient was stratified to the TRA group. If attempted CTO PCI used only femoral access (single or dual arterial access), the patient was categorized to the TFA group.

The temporal trend analysis was modeled with overall access (radial or femoral) as the outcome. The primary outcome for the analytic cohort was a safety composite of procedural complications, access site complications, in-hospital bleeding, or 30-day mortality. Procedural complications included death, stroke, emergency cardiac surgery, cardiogenic shock, coronary perforation, coronary dissection, emergent escalation of mechanical circulatory support, and cardiac tamponade. Access site complications included dissection, perforation, hematoma, retroperitoneal hemorrhage, limb ischemia, or vascular injury requiring surgical repair. In-hospital bleeding was defined using the presence of Bleeding Academic Research Consortium^32^ 3a bleeding on the date of the CTO PCI. The successful treatment of a CTO lesion was defined by either placement of any stent with a poststenosis of ≤30% or no stent placement and a poststenosis of ≤50%. The secondary outcome of procedural success was defined by the successful treatment of all CTO lesions within a vessel without any procedural complications.

### Statistical analysis

To examine temporal trends in CTO PCI radial access site use, the frequency was plotted yearly in calendar year 2010-2017. Nested logistic regression models were used to perform a likelihood ratio test with TRA as the outcome. To assess the composite primary outcome and procedural success after CTO PCI, propensity score matching methods were used. For each patient, a propensity score was estimated as the conditional probability that they would have had TRA using a multivariable logistic regression model. Variables in the propensity model included patient and procedural characteristics that were considered potential confounders for procedural success,^1^ such as age, body mass index, ethnicity, previous MI, previous PCI, previous coronary artery bypass graft (CABG), peripheral arterial disease, type 2 diabetes, hypertension, hyperlipidemia, tobacco use, creatinine, use of P2Y12 inhibitors before CTO PCI, number of overall lesions treated, bifurcation lesions, and calcified lesions. Common support for propensity scores for the femoral and radial groups was confirmed graphically with both a histogram and a box plot. After determining patients’ propensity scores, each patient in the TRA group was matched to a patient in the TFA group. Matching was based on patients’ propensity scores, with caliper width of 0.3 times the SD of the logit of the propensity score, VA site, and time. Patients in the TRA group were preferentially matched to patients in the TFA group within the same facility and calendar year. After obtaining the cohort of patients and their matched counterparts, propensity score distributions between the 2 access site groups were again graphed, and covariate balance was assessed using standardized differences. Standardized differences <0.1 were considered not significant and indicated a good balance.

Finally, a standard logistic regression model was constructed using our matched cohort to estimate the association between access site on primary and secondary outcomes. Sensitivity analyses were completed with the same propensity score calculation and matching steps but with different selection criteria regarding which VA sites to exclude. The sensitivity analyses included the following: (1) the same exclusions as in the primary analysis with the addition of excluding any CTO PCI that had dual, mixed arterial access, (2) only excluding VA sites with >90% femoral access (keeping sites with >90% radial), and (3) excluding “extreme” sites that had >80% femoral or >80% radial access.

All analyses were performed using R version 3.6.1 with matching preformed with the Matching package. *P* values of <.05 were considered significant.

## Results

**Figure 1:**
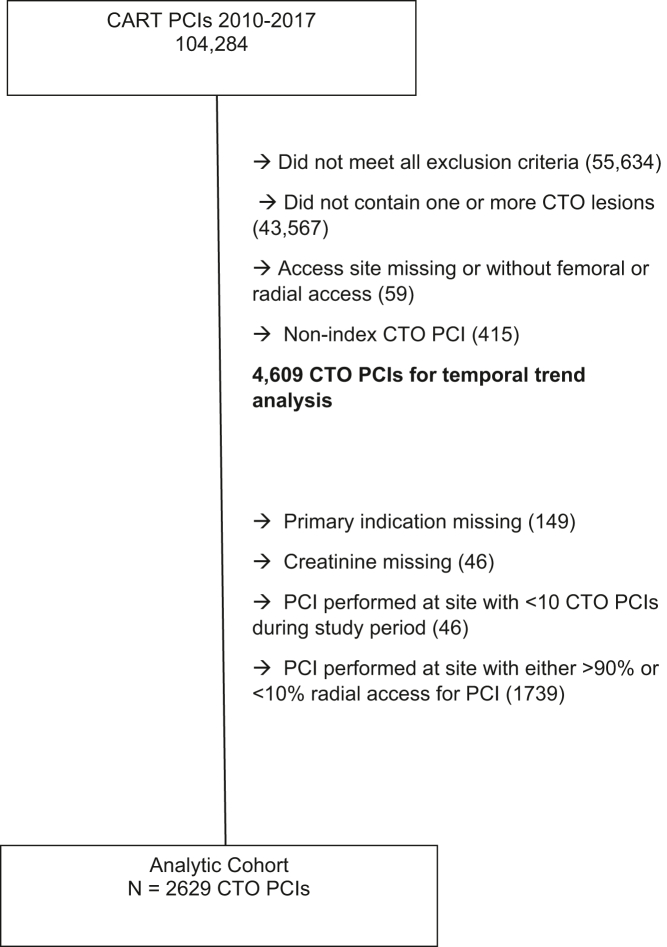
**CONSORT flow diagram of cohort construction.** The construction of the temporal trend and analytic cohorts from an initial query of the Clinical Assessment, Reporting, and Tracking System for Cardiac Catheterization Laboratories database. CART, Clinical Assessment, Reporting, and Tracking System; CTO, chronic total occlusion; PCI, percutaneous coronary intervention.

**Figure 2:**
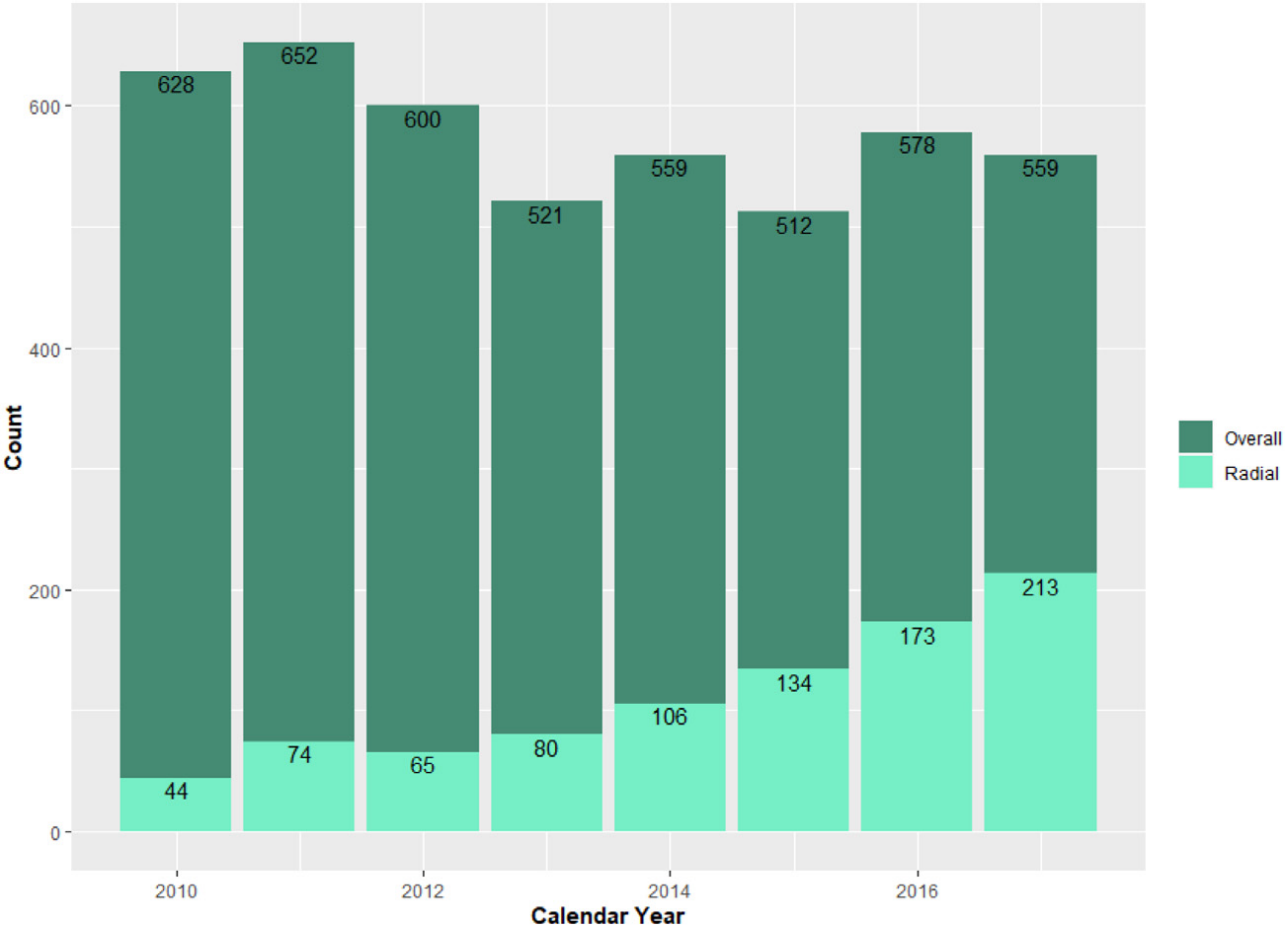
**Total number of attempted index CTO PCIs bycalendaryear.** CTO, chronic total occlusion; PCI, percutaneous coronary intervention.

**Figure 3:**
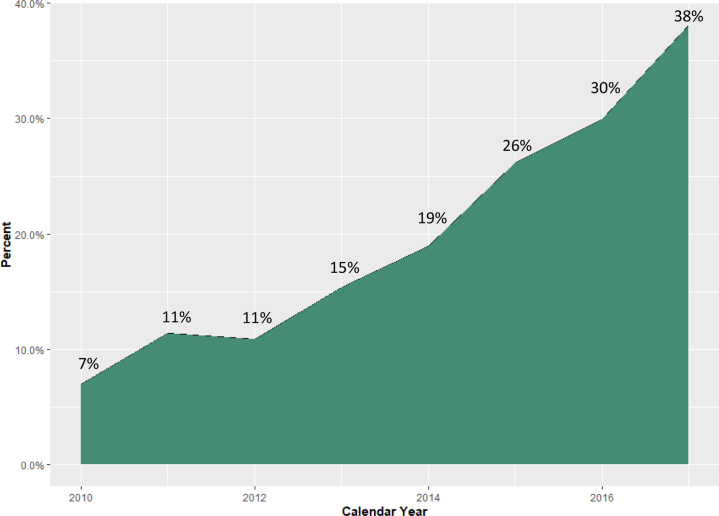
**Percent attempted index CTO PCIs with transradial access bycalendaryear**. CTO, chronic total occlusion; PCI, percutaneous coronary intervention.

The patient populations eligible for inclusion in our temporal trend cohort and analytic cohort are shown in Figure 1. Between January 1, 2010, and December 31, 2017, 4609 attempted CTO PCIs meeting inclusion and exclusion criteria were identified from CART data. The volume and proportion of attempted CTO PCI using TRA increased over time, from 7% in 2010 to 38% in 2017 (*P*_trend_ < .01) (Figures 2, 3, and Central Illustration).

Baseline patient and procedural characteristics for the temporal trend cohort are summarized in Table 1. Of the 4609 patients included, 3720 patients had TFA (81%) for attempted CTO PCI and 889 had TRA (19%). Patient demographics and comorbidities were similar between the 2 groups, with some notable exceptions. Patients in the TFA group were more likely to be White and have a history of previous MI, previous PCI, and previous CABG compared with patients in the TRA group. Conversely, patients in the TRA group were more likely to have a history of tobacco use. Lesion characteristics are summarized in Table 2. A greater percentage of CTO lesions in the TFA group was calcified and >20.0 mm in length. Atherectomy and intravascular ultrasound were more likely to be used in the TFA group. Both TRA and TFA groups had similar mean fluoroscopy times for attempted CTO PCI although contrast utilization was higher in the TFA group (274.0 mL vs 244.0 mL, *P* < .01).

**Table 1:** Baseline patient characteristics in the temporal trend cohort.

	All patients (N = 4609)	Access site		*P*
		TFA (n = 3720)	TRA (n = 889)	
Age, y	65.6 ± 8.3	65.6 ± 8.3	65.4 ± 8.2	.50
Body mass index, kg/m^2^	30.9 ± 5.6	30.9 ± 5.5	31.0 ± 5.9	.68
Male sex	4567 (99.1%)	3690 (99.2%)	877 (98.7%)	.13
Race				< .01
White	3937 (85.4%)	3212 (86.3%)	725 (81.6%)	
Black	553 (12.0%)	418 (11%)	135 (15.2%)	
Other	119 (2.6%)	90 (2.4%)	29 (3.3%)	
Medical history				
Previous myocardial infarction	2100 (45.6%)	1723 (46.3%)	377 (42.4%)	.04
Previous coronary artery bypass grafting	1228 (26.6%)	1043 (28.0%)	185 (20.8%)	<.01
Previous PCI	2451 (53.2%)	2012 (54.1%)	439 (49.4%)	.01
Diabetes mellitus	2248 (48.8%)	1827 (49.1%)	421 (47%)	.35
Peripheral vascular disease	1008 (21.9%)	801 (21.5%)	207 (23.3%)	<.001
Hypertension	4226 (91.7%)	3401 (91.4%)	825 (92.8%)	.18
Hyperlipidemia	4340 (94.2%)	3495 (94.0%)	845 (95.1%)	.21
History of smoking	3050 (66.2%)	2407 (64.7%)	643 (72.3%)	<.01
Chronic renal insufficiency				.02
CKD III	957 (21.0%)	775 (21.0%)	182 (20.8%)	
CKD IV	41 (0.9%)	35 (1.0%)	6 (0.7%)	
CKD V/ESRD	82 (1.8%)	76 (2.1%)	6 (0.7%)	
GFR, mL/min	75.2 ± 23.3	74.7 ± 23.3	77.1 ± 23.3	<.01
P2Y12 use within 90 d before PCI	2191 (47.5%)	1778 (47.8%)	413 (46.5%)	.47
P2Y12 use within 90 d after PCI	3896 (84.5%)	3163 (85.0%)	733 (82.5%)	.06
Primary PCI indication				<.01
Arrhythmia	1 (0.0%)	0 (0.0%)	1 (0.1%)	
Asymptomatic	126 (2.8%)	107 (3.0%)	19 (2.2%)	
Cardiomyopathy	3 (0.1%)	1 (0.0%)	2 (0.2%)	
Chest pain	215 (4.8%)	166 (4.6%)	49 (5.6%)	
Other	345 (7.7%)	265 (7.4%)	80 (9.1%)	
Positive functional study	3 (0.1%)	1 (0.0%)	2 (0.2%)	
Stable angina	3757 (84%)	3037 (85%)	720 (82%)	
Valvular heart disease	10 (0.2%)	7 (0.2%)	3 (0.3%)	

Values are mean ± SD or n (%).

CKD, chronic kidney disease; ESRD, end-stage renal disease; GFR, glomerular filtration rate; PCI, percutaneous coronary intervention; TFA, transfemoral access; TRA, transradial access.

**Table 2:** Baseline procedural and lesion characteristics in the temporal trend cohort.

	All patients (N = 4609)	Access site		*P*
		TFA (n = 3720)	TRA (n = 889)	
Single access site	3193 (69.3%)	2544 (68.4%)	649 (73.0%)	<.01
Contrast volume, mL	268 ± 138	274 ± 138	244 ± 133	<.01
Fluoroscopy time, min	38.5 ± 27.0	38.7 ± 27.0	37.5 ± 27.0	.24
CTO lesion count	1.404 ± 0.90	1.09 ± 0.34	1.05 ± 0.27	<.01
Target vessel				.71
Left anterior descending artery	1255 (28.1%)	1015 (28.2%)	240 (27.4%)	
Left circumflex artery	1186 (26.5%)	952 (26.5%)	234 (26.7%)	
Left main coronary artery	17 (0.4%)	14 (0.4%)	3 (0.3%)	
Right coronary artery	1948 (43.5%)	1559 (43.3%)	389 (44.5%)	
Calcification of CTO lesion	1187 (25.8%)	991 (26.6%)	196 (22.0%)	<.01
Presence of bifurcation CTO lesion	245 (5.3%)	192 (5.2%)	53 (6.0%)	.34
Any CTO lesion with length >20.0 mm	2135 (59.5%)	1783 (61.3%)	352 (52.0%)	<.01
Intravascular ultrasound for CTO PCI	495 (10.7%)	422 (11.3%)	73 (8.2%)	<.01
Atherectomy for treatment of CTO lesion	303 (7.4%)	280 (8.4%)	23 (3.0%)	<.01

Values are mean ± SD or n (%).

CTO, chronic total occlusion; PCI, percutaneous coronary intervention; TFA, transfemoral access; TRA, transradial access.

**Figure 4:**
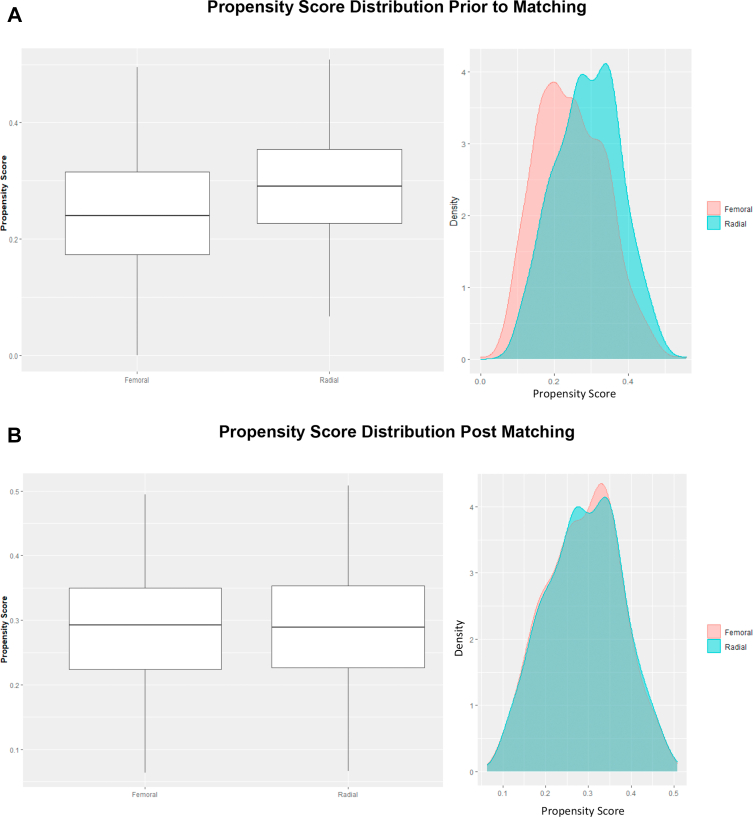
**Propensity score distributions**. (**A**) Propensity score distribution prior to matching. (**B**) Propensity score distribution after matching.

After excluding low-volume CTO sites, VA sites with >90% femoral or radial access, and those with missing creatine or primary indication, the primary analytic cohort consisted of 2629 attempted CTO PCIs across 36 VA sites, with 676 of these CTO PCIs having TRA. Patient and procedural characteristics for the primary analytic cohort are summarized in Table 3. Before matching, patients in the TFA group were more likely to be White, have a history of previous CABG, and have calcified CTO lesions compared with patients in the TRA group. Patients in the TRA group were more likely to be tobacco users. After propensity matching, the analytic cohort included 1342 attempted CTO PCIs, with equal numbers in the TRA and TFA groups. The propensity-matched analytic cohort showed improved covariate balance and improved distribution of propensity scores (Table 4 and Figure 4), with covariate imbalance remaining only for previous CABG and calcification.

**Table 3:** Patient and procedural characteristics in the analytic cohort prior to matching.

	All patients (N = 2629)	Access site		Standardized mean difference
		TFA (n = 1953)	TRA (n = 676)	
Age, y	65.7 ± 8.3	65.7 ± 8.4	65.5 ± 8.2	0.02
Body mass index, kg/m^2^	30.8 ± 5.5	30.7 ± 5.3	31.0 ± 5.8	
Male sex	2602 (99.0%)	1934 (99.0%)	668 (98.8%)	0.020
White race	2280 (86.7%)	1719 (88.0%)	561 (83.0%)	0.143
Medical history				
Previous myocardial infarction	1192 (45.3%)	896 (45.9%)	296 (43.8%)	0.042
Previous coronary artery bypass grafting	717 (27.3%)	578 (29.6%)	139 (20.6%)	0.210
Previous PCI	1366 (52.0%)	1026 (52.5%)	340 (50.3%)	0.045
Diabetes mellitus	1275 (48.5%)	956 (49.0%)	319 (47.2%)	0.035
Peripheral vascular disease	602 (22.9%)	448 (22.9%)	154 (22.8%)	0.004
Hypertension	2422 (92.1%)	1789 (91.6%)	633 (93.6%)	0.078
Hyperlipidemia	2460 (93.6%)	1818 (93.1%)	642 (95.0%)	0.080
History of smoking	1712 (65.1%)	1230 (63.0%)	482 (71.3%)	0.178
Creatinine, mg/dL	1.16 ± 0.66	1.17 ± 0.70	1.12 ± 0.53	0.087
P2Y12 use within 90 d before PCI	1246 (47.4%)	926 (47.4%)	320 (47.3%)	0.002
Primary PCI indication				0.121
Arrhythmia	0 (0.0%)	0 (0.0%)	0 (0.0%)	
Asymptomatic	78 (3.0%)	61 (3.1%)	17 (2.5%)	
Cardiomyopathy	1 (0.0%)	1 (0.1%)	0 (0.0%)	
Chest pain	126 (4.8%)	93 (4.8%)	33 (4.9%)	
Other	193 (7.3%)	130 (6.7%)	63 (9.3%)	
Positive functional study	1 (0.0%)	1 (0.1%)	0 (0.0%)	
Stable angina	2223 (84.6%)	1663 (85.2%)	560 (82.8%)	
Valvular heart disease	7 (0.3%)	4 (0.2%)	3 (0.4%)	
CTO lesion count	1.09 ± 0.34	1.10 ± 0.36	1.05 ± 0.27	0.141
Calcification of CTO lesion	826 (31.4%)	688 (35.2%)	138 (20.4%)	0.335

Values are mean ± SD or n (%).

CTO, chronic total occlusion; PCI, percutaneous coronary intervention; TFA, transfemoral access; TRA, transradial access.

**Table 4:** Patient and procedural characteristics in the analytic cohort after matching.

	Access site			
	Overall (n = 1342)	TFA (n = 671)	TRA (n = 671)	Standardized mean difference
Age, y	65.79 ± 8.2	65.9 ± 8.1	65.5 ± 8.2	0.059
Body mass index, kg/m^2^	31.0 ± 5.6	31.0 ± 5.3	31.0 ± 5.8	0.003
Male sex	1327 (98.9%)	664 (99.0%)	663 (98.8%)	0.014
White race	1122 (83.6%)	564 (84.1%)	558 (83.2%)	0.024
Medical history				
Previous myocardial infarction	618 (46.1%)	324 (48.3%)	294 (43.8%)	0.090
Previous coronary artery bypass grafting	323 (24.1%)	185 (27.6%)	138 (20.6%)	0.164
Previous PCI	700 (52.2%)	363 (54.1%)	337 (50.2%)	0.078
Diabetes mellitus	650 (48.4%)	333 (49.6%)	317 (47.2%)	0.048
Peripheral vascular disease	308 (23.0%)	155 (23.1%)	153 (22.8%)	0.007
Hypertension	1257 (93.7%)	629 (93.7%)	628 (93.6%)	0.006
Hyperlipidemia	1280 (95.4%)	643 (95.8%)	637 (94.9%)	0.043
History of smoking	958 (71.4%)	481 (71.7%)	477 (71.1%)	0.013
Creatinine, mg/dL	1.13 ± 0.58	1.14 ± 0.62	1.12 ± 0.53	0.087
P2Y12 use within 90 d before PCI	659 (49.1%)	341 (50.8%)	318 (47.4%)	0.069
Primary PCI indication				0.090
Arrhythmia	0 (0.0%)	0 (0.0%)	0 (0.0%)	
Asymptomatic	37 (2.8%)	20 (3.0%)	17 (2.5%)	
Cardiomyopathy	0 (0.0%)	0 (0.0%)	0 (0.0%)	
Chest pain	64 (4.8%)	31 (4.6%)	33 (4.9%)	
Other	133 (9.9%)	73 (10.9%)	60 (8.9%)	
Positive functional study	0 (0.0%)	0 (0.0%)	0 (0.0%)	
Stable angina	1104 (82.3%)	546 (81.4%)	558 (83.2%)	
Valvular heart disease	4 (0.3%)	1 (0.1%)	3 (0.4%)	
CTO lesion count	1.06 ± 0.29	1.07 ± 0.30	1.06 ± 0.27	0.052
Calcification of CTO lesion	250 (18.6%)	112 (16.7%)	138 (20.6%)	0.100

Values are mean ± SD or n (%).

CTO, chronic total occlusion; PCI, percutaneous coronary intervention; TFA, transfemoral access; TRA, transradial access.

**Central Illustration:**
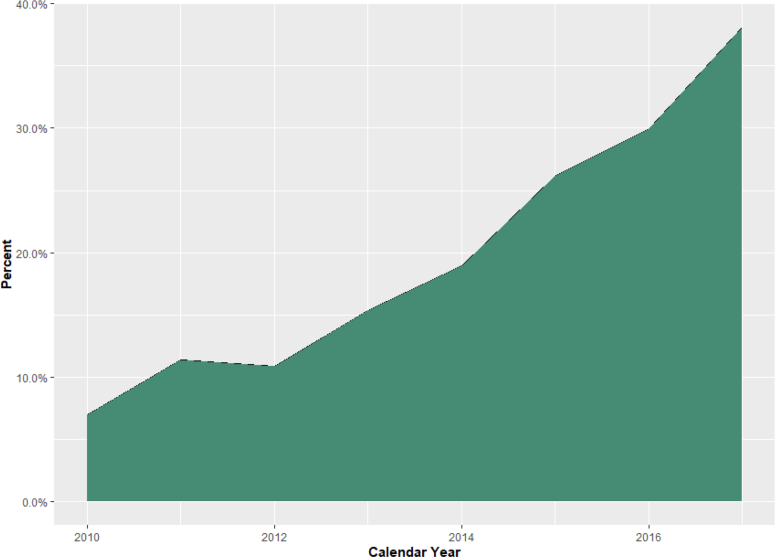
Transradial access for chronic total occlusion percutaneous coronary interventions has significantly increased in the Veteran population in the contemporary era.

In the propensity-matched cohort, logistic regression models were used for the composite primary safety outcome and secondary outcome of procedural success. The primary composite safety outcomes (procedural complications, access site complications, in-hospital bleeding, or 30-day mortality) were observed in 3.3% of patients in the TRA group and 4.0% of patients in the TFA group (*P* = .47). Individual components of the primary composite outcome are reported in Table 5. Procedural success was achieved in 66.6% of patients in the TRA group and 65.7% of patients in the TFA group (*P* = .74), as shown in Supplemental Table S1. Sensitivity analyses were performed, with similar results to the primary analysis regarding both safety and efficacy outcomes, as summarized in Supplemental Tables S1 and S2.

**Table 5:** Composite primary outcome components and secondary outcome in the primary analysis.

Outcome	Access site		*P*
	TFA (n = 671)	TRA (n = 671)	
Any access site complication	5 (0.7%)	1 (0.1%)	.10
Any procedural complication	5 (0.7%)	9 (1.3%)	.28
BARC 3a bleeding	16 (2.4%)	8 (1.2%)	.10
Death within 30 d of PCI procedure date	4 (0.6%)	5 (0.7%)	.74
Composite primary end point	27 (4.0%)	22 (3.3%)	.47
Procedural success	423 (65.7%)	428 (66.6%)	.74

Values are n (%).

BARC, Bleeding Academic Research Consortium; PCI, percutaneous coronary intervention; TFA, transfemoral access; TRA, transradial access.

## Discussion

Our study highlights the increasing use of TRA for CTO PCI over time, rising from 7% in 2010 to 38% in 2017 (*P*_trend_ < .01). The most likely factors responsible for this trend are advances in device design that allow for increasingly complex PCI using TRA and the rapid increase in overall TRA use by the interventional cardiology community in the United States.

The evolution of device technology has helped facilitate the increasing prevalence of TRA for complex PCI, including CTO lesions. Improvements in specialized coronary guide wires and low-profile microcatheters have allowed operators to use 6F catheter systems to approach CTO lesions with numerous strategies, including antegrade wire escalation, antegrade dissection/reentry, and retrograde lesion crossing. Furthermore, 6F guide extension catheters can be used to enhance support when necessary. The development of sheathless transradial guide wires has allowed for larger bore access using TRA, which may be required for additional guide catheter support, trapping, or specific reentry strategies such as subintimal transcatheter withdrawal.

Moreover, the increasing rates of TRA for CTO PCI observed in this study reflects the overall increasing comfort of U.S. operators with the transradial approach. The proportion of all PCI through the transradial approach has increased dramatically over the last 15 years.^1^, ^2^, ^3^ The uptake has been driven by continued data supporting reduced bleeding and vascular complications for TRA,^4^, ^5^, ^6^, ^7^, ^8^, ^9^, ^10^, ^11^ demonstrations and discussions of complex TRA procedures during national scientific meetings, and numerous transradial proctorship programs, which have allowed operators to ascend the learning curve required for proficiency.

Our study demonstrates that transradial CTO PCI has a safety profile similar to the transfemoral approach without compromising technical success rates. Previous retrospective, registry-based studies have shown a significant reduction in major bleeding with TRA for CTO PCI: rates of 0.2% to 0.8% and 0.8% to 1.9% were observed with TRA and TFA, respectively.^20^^,^^22^^,^^23^ Rates of in-hospital bleeding, defined as Bleeding Academic Research Consortium 3a bleeding in our study, were 1.2% in the TRA group and 2.4% in the TFA group (*P* = .10). Although there were fewer events in the TRA group, our study is underpowered to detect differences between the groups because there were only 24 events in the entire propensity-matched cohort. Routine use of safe femoral access practices, including both fluoroscopic and ultrasound guidance for femoral artery puncture, routine femoral angiography, and placement of vascular closure devices, have been important for minimizing vascular access complications and bleeding after transfemoral PCI, especially in the case of elective planned CTO PCI procedures. Because the use of TRA for CTO PCI is associated with similar safety and procedural success to TFA, it should be considered owing to patient preference, increased patient comfort, and potential for the same day discharge.^33^

CTO complexity may influence an operator’s decision regarding the number and location of access sites. This selection bias was evident in our unadjusted baseline characteristics, in which patients in the TFA group were more likely to have CTO lesions that were calcified and >20.0 mm in length compared with patients in the TRA group. Although our database limits our ability to calculate a complete J-CTO score, the presence of calcium and lesion length will increase the likelihood that the TFA group had higher J-CTO scores. We used propensity matching to address these differences in baseline lesion characteristics and after matching, the primary analysis and sensitivity analyses showed similar rates of technical success with TRA and TFA for attempted CTO PCI. These data are consistent with previous retrospective studies that have also demonstrated no difference in technical success with either TRA or TFA for CTO PCI, although the definition of technical success varied between studies.^22^^,^^23^

The current study has limitations that must be considered when interpreting the data. As discussed, a retrospective observational study has inherent limitations; by performing a propensity-matched analysis of the primary and secondary outcomes, we sought to mitigate confounding associated with observational analyses. Second, if any radial access was used for a given case, we stratified that patient to the TRA group to determine the effect of TRA on outcomes for CTO PCI. Hence, patients with mixed TFA and TRA were part of the TRA group, which could potentially confound the outcome data. To address this, a sensitivity analysis excluding patients with mixed arterial access was performed, which showed similar odds ratios for the primary and secondary outcomes. Third, our veteran study population was predominantly male patients, which may limit the extrapolation of our data to more diverse populations. We excluded VA sites that performed >90% of cardiac catheterizations using the transradial or transfemoral approach to eliminate bias from sites with a clear predilection for a specific access route. Fourth, the CART database is not a dedicated registry for CTO PCI and has some inherent limitations for our study. It relies on the operator to identify a CTO lesion, which could be subjective in some cases. Procedural data collected in the CART do not capture CTO-crossing strategies or fully describe the CTO lesion complexity. To address the latter, we stratified CTO lesions by calcification, lengths >20.0 mm, and tortuosity. Finally, given the low overall event rate of the primary outcome components, our study was underpowered to detect a statistically significant difference in safety profile between the TRA and TFA groups.

## Conclusions

In conclusion, the proportion of TRA for CTO PCI has increased steadily over time. TRA for CTO PCI is associated with a similar safety profile to TFA without compromising procedural success. Hence, there is an opportunity to further increase the use of TRA. Future prospective and randomized studies are needed to determine the relative safety and efficacy of access site selection for CTO PCI.
