# Supplementary material for: Access Site Selection and Outcomes for Chronic Total Occlusion Percutaneous Coronary Interventions: Insights from the VA CART Program
Source: J Soc Cardiovasc Angiogr Interv. 2022 Aug 24;1(6):100440. doi: 10.1016/j.jscai.2022.100440 (PMC11308032; doi:10.1016/j.jscai.2022.100440)
Supplement: Supplemental Table S1 [file mmc1.docx]

**Table S1: Rates of the Primary Composite Endpoint in the Primary and Sensitivity Analyses**

|  | **Access Site** | |  |
| --- | --- | --- | --- |
| **Analysis Exclusions** | **TFA – no. (%)** | **TRA - no. (%)** | **P-value** |
| >90% radial & >90% femoral sites excluded (primary analysis) | 27 (4.0%) | 22 (3.3%) | 0.47 |
| >90% radial, >90% femoral & mixed access excluded | 17 (3.5%) | 14 (2.9%) | 0.58 |
| >90% femoral sites excluded | 28 (3.8%) | 23 (3.1%) | 0.48 |
| >80% radial & >80% femoral sites excluded | 22 (5.1%) | 14 (3.3%) | 0.17 |
