# Supplementary material for: Access Site Selection and Outcomes for Chronic Total Occlusion Percutaneous Coronary Interventions: Insights from the VA CART Program
Source: J Soc Cardiovasc Angiogr Interv. 2022 Aug 24;1(6):100440. doi: 10.1016/j.jscai.2022.100440 (PMC11308032; doi:10.1016/j.jscai.2022.100440)
Supplement: Supplemental Table S2 [file mmc2.docx]

**Table S2: Rates of the Secondary Endpoint (Procedural Success) in the Primary and Sensitivity Analyses**

|  | **Access Site** | |  |
| --- | --- | --- | --- |
| **Analysis Exclusions** | **TFA – no. (%)** | **TRA - no. (%)** | **P-value** |
| >90% radial & >90% femoral sites excluded (primary analysis) | 423 (65.7%) | 428 (66.6%) | 0.74 |
| >90% radial, >90% femoral & mixed access excluded | 323 (69.8%) | 313 (67.3%) | 0.42 |
| >90% femoral sites excluded | 468 (65.5%) | 476 (66.9%) | 0.58 |
| >80% radial & >80% femoral sites excluded | 268 (66.0%) | 282 (68.6%) | 0.43 |
